# Overexpression of the *Artemisia* Orthologue of ABA Receptor, AaPYL9, Enhances ABA Sensitivity and Improves Artemisinin Content in *Artemisia annua* L

**DOI:** 10.1371/journal.pone.0056697

**Published:** 2013-02-20

**Authors:** Fangyuan Zhang, Xu Lu, Zongyou Lv, Ling Zhang, Mengmeng Zhu, Weiming Jiang, Guofeng Wang, Xiaofen Sun, Kexuan Tang

**Affiliations:** Plant Biotechnology Research Center, School of Agriculture and Biology, Shanghai Jiao Tong University, Shanghai, People's Republic of China; RIKEN Plant Science Center, Japan

## Abstract

The phytohormone abscisic acid (ABA) plays an important role in plant development and environmental stress response. In this study, we cloned an ABA receptor orthologue, AaPYL9, from *Artemisia annua* L. *AaPYL9* is expressed highly in leaf and flower. AaPYL9 protein can be localized in both nucleus and cytoplasm. Yeast two-hybrid assay shows AaPYL9 can specifically interact with AtABI1 but not with AtABI2, AtHAB1 or AtHAB2. ABA can enhance the interaction between AaPYL9 and AtABI1 while AaPYL9-89 Pro→Ser and AaPYL9-116 His→Ala point mutations abolishes the interaction. BiFC assay shows that AaPYL9 interacts with AtABI1 in nucleus *in planta*. Transgenic *Arabidopsis* plants over-expressing *AaPYL9* are more sensitive to ABA in the seed germination and primary root growth than wild type. Consistent with this, ABA report genes have higher expression in *AaPYL9* overexpressing plants compared to wild type after ABA treatment. Moreover, overexpression of *AaPYL9* in *A. annua* increases not only drought tolerance, but also artemisinin content after ABA treatment, with significant enhancement of the expression of key genes in artemisinin biosynthesis. This study provides a way to develop *A. annua* with high-yielding artemisinin and high drought resistance.

## Introduction

The phytohormone abscisic acid (ABA) is a key regulator, involved in different plant developmental processes such as seed germination, root elongation, and bud dormancy [1], as well as in plant responses to abiotic stresses including drought, salt, osmotic and cold stress [2], [3]. ABA acts through a complex signaling cascade to induce changes in gene expression and in adaptive physiological responses [4]–[6]. Many ABA-regulated genes have been identified, and genome-scale analyses indicate that a large number of genes are responsive to ABA in *Arabidopsis* [4], [7]–[9]. Some of the ABA signaling components associated with germination have been characterized, including guanine nucleotide-binding proteins (G proteins), kinases, phosphatases, protein degradation pathways, transcription factors, and secondary messengers [6], [10]–[17]. Several reviews of ABA bio-synthesis and signaling have been published recently [18]–[20]. A breakthrough in 2009 in ABA signaling is that, members of the PYR1/PYL/RCAR family of proteins are proved to be ABA receptors in the cytoplasm and nucleus [21]–[23]. In *Arabidopsis*, the PYR1/PYL/RCAR family have 14 members (PYR1 and PYL1-13), which previously were classified as plant pathogenesis-related proteins of class 10 (PR-10) [24]. The discovery of ABA receptors greatly promotes us to gain an intimate knowledge about structural basis of ABA perception and signaling in more detail. Several elegant crystallographic studies described the interaction between the ABA molecule and PYR1/PYL/RCAR receptors, and the type 2C protein phosphatases (PP2C) [23], [25]–[30]. In general, the structure of PYL/RCARs proteins changes after bound by ABA, and such an interaction locks the ABA-binding pocket of the receptor. Both results demonstrate that ABA-bounded PYL/RCARs interact with clade A PP2Cs and inhibits their phosphatase activity, resulting in an activation of subclass III SnRK2s in the presence of ABA. Consequently, ABA signaling is activated and the ABA-responsive genes, such as *RD29* and *RAB18* are induced [31]. In plants, the ABA receptor PYR/PYL/RCAR family is evolutionarily conserved [19], [32]. Klingler et al. demonstrated on the basis of the available genome sequences of several crops levels of family diversity for the PYR/PYL/RCAR proteins similar to that of *Arabidopsis* [19]. Umezawa et al. also analyzed the PYR/PYL/RCAR in bryophyte, lycophyte and angiosperm, revealed bryophyte has four ABA receptor homologues and vascular plants have more than 10 homologues.

Malaria is a global health problem with more than one billion people living in areas with a high risk of the disease [33]. Artemisinin, isolated from traditional Chinese herb *Artemisia annua* L. (qinghao), is a sesquiterpene lactone endoperoxide that provides the basis for effective treatments of malaria, especially for the cerebral and the chloroquine-resistant forms of this disease [34]. Since 2001, the World Health Organization (WHO) recommended the artemisinin based combination therapies (ACTs) to be the most effective and safe treatments. Besides the antimalarial activity, artemisinin has also been reported to antiviral [35], anticancer [36] and antischistosomal activities [37]. At present, *A. annua* plant is still the only commercial source of artemisinin. Unfortunately, only very low amounts of artemisinin can be isolated from the aerial parts of *A. annua* (between 0.01 and 0.6% dry weight) [38], [39].

Interestingly, the content of artemisinin is induced by exogenous ABA [40]. And in *Arabidopsis*, secondary metabolic processes are also enriched in both guard cell and leaf ABA-induced genes [41]. The understanding of the ABA signaling pathway in *Artemisia* will provide the basis for artimisinin genetic engineering. Moreover, the enhancement of ABA sensitivity of plants has been proved to be an efficient way to increase biomass under abiotic stress, including drought [42]–[44]. As ABA receptors, PYR/PYL/RCAR will be of great value in the increase of ABA sensitivity. However, to our knowledge, until now, no ABA receptor from *A. annua* has been cloned and functionally characterized.

In the present study, we report that an ABA receptor, AaPYL9, was successfully cloned from *A. annua* and functionally studied in *A. annua* as well as in model plant *Arabidopsis*. *AaPYL9* codes for a PYR/PYL/RCAR family protein, with high similarity to *Arabidopsis* PYL9. Yeast two-hybrid and BiFC assays show that AaPYL9 interacts with AtABI1 in yeast and *in planta*, while AaPYL9-86 Pro→Ser and AaPYL9-116 His→Ala point mutations abolishes the interaction. Overexpression of *AaPYL9* (*AaPYL9* OX) in *Arabidopsis* greatly reduced the seed germination rate under various abiotic stress treatments (ABA, osmotic and salt). Moreover, the *AaPYL9* OX *Arabidopsis* showed reduced root elongation under ABA treatment during seedling growth. Further expression analysis showed that overexpression of *AaPYL9* in *Arabidopsis* increased the ABA sensitivity in early seedling growth stage. Furthermore, overexpression *AaPYL9* in *Artemisia* increased drought tolerance and improved artemisinin content. The cloning and functional analysis of *A. annua* ABA receptors provides a basis for further studying the roles of the ABA receptor on the regulation of artemisinin biosynthesis in *A. annua* plant.

## Results

### Isolation of *AaPYL9* cDNA

**Figure 1:**
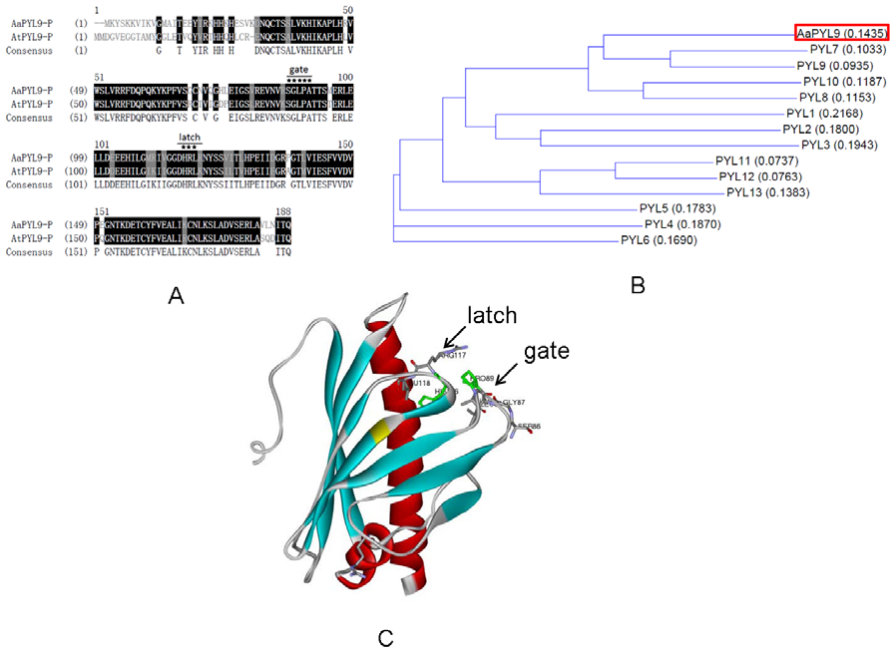
Sequence analysis of *AaPYL9*. A: Sequence alignment of AaPYL9 protein with PYL9 in *Arabidopsis*. Positions with identical amino acid residues are highlighted in black, while similar amino acid residues are colored in gray. B: Phylogenetic tree showing the relationship between AaPYL9 and other PYR/PYL/RCAR proteins. The tree presented here is a Neighbor–Joining tree based on amino acid sequence alignment. The numbers next to each node give bootstrap values for 1000 replicates. C: Deduced three-dimensional structure of START domains indicating the structure of AaPYL9 is similar with AtPYL9. The gate residue and latch residue are shown, and gate residue P89 and latch residue H116 are shown in green.

To study the biosynthesis of artemisinin, a cDNA library of *A. annua* was constructed using leaf as the source of mRNA. In total, 2,000 randomly picked clones were sequenced. By blast searching the 2,000 sequences, *AaPYL9* was identified that encoded PYR/PYL/RCAR family member (Fig. 1A, 1B). AaPYL9 protein exhibited 72.6% sequence identity with *Arabidopsis* AtPYL9 protein (Fig. 1A). To gain insights into the function of *AaPYL9*, the expression pattern of *AaPYL9* was initially studied by quantitative real-time PCR. *AaPYL9* was expressed in whole plant, and the highest in leaf (Fig. S1A). We also analyzed the expression pattern of endogenous *AaPYL9* gene in *A. annua* under ABA, drought and NaCl treatment (Fig. S1B–S1D). There is no notable change when treated with 10 µM ABA, drought and 200 mM NaCl (Fig. S1B–S1D).

### Identification of AaPYL9-interacting proteins in Arabidopsis by yeast two-hybrid screening

**Figure 2:**
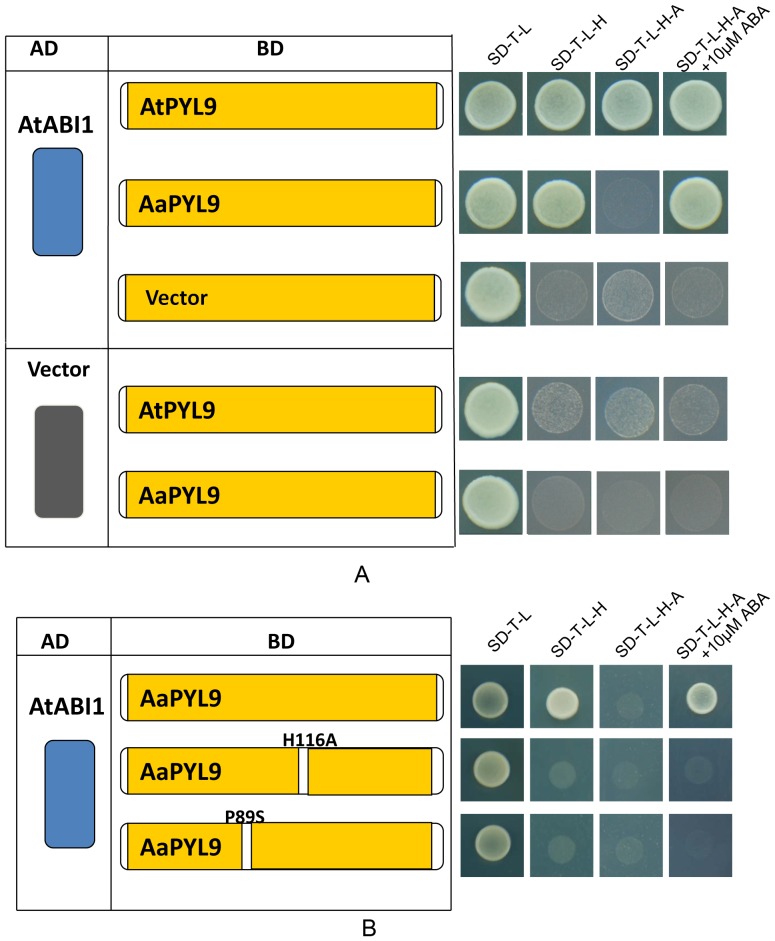
Yeast two-hybrid assay for AaPYL9-interacting proteins. Two-hybrid interaction test of AaPYL9 (native protein and two different point mutations) with AtABI1 (AT4G26080). Left columns show bait and prey plasmids. The photograph on the right shows the results of yeast two-hybrid assays on control medium that lacked leu, trp (SD/-T-L) and selection plate that lacked trp, leu, his (SD/-T-L-H), lacked trp leu, his, ade (SD/-T-L-H-A), and lacked trp leu, his, ade supplemented with 10 µM ABA(SD/-T-L-H-A+10 µM ABA). A: PYL9s from *Arabidopsis* and *Artemisia* were constructed as binding-domain (BD) fusion proteins, and tested their interactions with activation domain (AD)-fused AtABI1 with the Y2H assays by supplemented with or without ABA. Yeast strain expresses AD-AtABI1 and BD-AtPYL9 could grew well on SD/-T-L-H-A medium and SD/-T-L-H-A+10 µM ABA medium. However, Yeast cells harbouring AD-AtABI1 and BD-AaPYL9 only grew well on SD/-T-L-H-A+10 µM ABA medium. The empty vector used as negative control. B: Effect of point mutations on the interaction between AaPYL9 and AtABI1 in the yeast two-hybrid assay. (Left) Diagram of point mutation sites, H116A indicates a His to Ala mutation at the 116 latch residue sites. P89S indicates a Pro to Ser mutation at the 89 gate residue sites. (Right) The mutations of AaPYL9 were fused with the binding-domain and introduced into yeast with the AD-AtABI1 construct. The growth on selective medium was shown. Yeast expresses BD-AaPYL9H116A and AD-AtABI1 could not grew on all kinds of mediums except SD/-T-L. Yeast cells harbouring BD-AaPYL9P89S and AD-AtABI1 have similar results.

In a search of possible AaPYL9-interacting proteins in *Arabidopsis*, a yeast two-hybrid screening was performed. AaPYL9 was fused to the Gal4 activation domain (AD domain) while four PP2C-A protein, AtABI1, AtABI2, AtHAB1 and AtHAB2, were fused to the Gal4 DNA binding domain (BD domain). Only AtABI1 showed interaction with AaPYL9 and adding ABA can increase the interaction (Fig. 2, S2). AD-AtPYL9 could interact constitutively with AtABI1, however, AaPYL9-AtABI1 interaction behaves as an ABA responsive interaction. Previous research showed that ABA receptor PYR/PYL/RCAR family proteins had a gate–latch–lock mechanism underlying ABA signaling [25], [29]. Two mutations were made in the gate region (P89S) and latch region (H116A), both of the mutations reduced the level of interaction between AaPYL9 and AtABI1 (Fig. 2).

### Subcellular localization of the AaPYL9 protein

**Figure 3:**
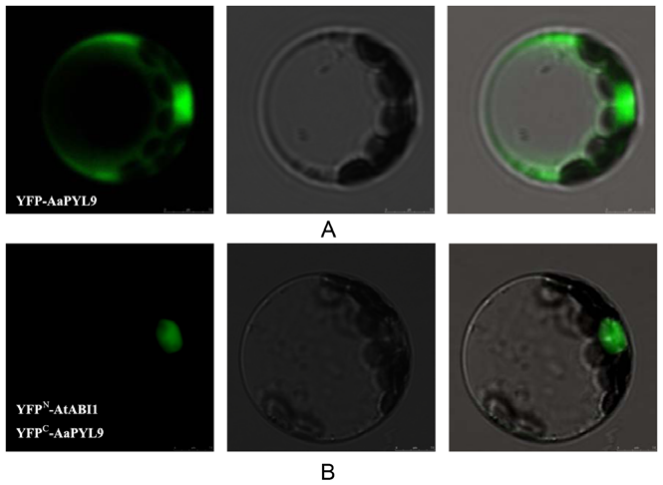
Subcellular localization of AaPYL9 and the protein physically interaction between AaPYL9 and AtABI1 *in planta*. A: subcellular localization of YFP fusions in transiently transformed *Arabidopsis* protoplasts. Confocal microscopy slides of cell sections show the AaPYL9 protein is located in the cytoplasm and nucleus. B: BiFC experiments show interaction between AaPYL9 and AtABI1 in *Arabidopsis* protoplasts. The localization of the interaction between YFP^C^-AaPYL9 and YFP^N^-AtABI1 was in the nucleus as shown by confocal microscopy images. The left column: YFP channel; The middle column: bright-field; The right column: merged picture.

To investigate the potential site of action of AaPYL9 and its interacting partner within the cell, the subcellular localization of these proteins was investigated. YFP protein was fused in frame to the full-length AaPYL9 protein (YFP-AaPYL9) and the fusion protein was driven under the control of the 35S promoter of Cauliflower mosaic virus (CaMV). *Arabidopsis* protoplasts transformed with an expression plasmid for the 35S::YFP-AaPYL9 exhibited YFP fluorescence in both nucleus and cytoplasm (Fig. 3A), as expected for a small soluble protein. These results showed that AaPYL9 can be localized in nucleus and cytoplasm, is a nucleocytoplasmic protein.

### *In planta* interaction between AaPYL9 and AtABI1

The protein interaction between AaPYL9 and the AtABI1 proteins was further confirmed in plant cells by bimolecular fluorescence complementation (BiFC). AaPYL9 was translationally fused to the C-terminal region of the yellow fluorescent protein (YFP^c^) in the pEG201-YC vector. Moreover, AtABI1 was translationally fused to the N-terminal region of the yellow fluorescent protein (YFP^N^) in the pEG202-YN vector. The constructs were co-transfected into *Arabidopsis* protoplasts cells, and as a result, fluorescence was observed in the nucleus of protoplasts cells (Fig. 3B). These data support the yeast two-hybrid assay and the subcellular localization, providing evidence for a physical interaction between AaPYL9 and AtABI1 proteins.

### Overexpression of AaPYL9 in *Arabidopsis* confers ABA-hypersensitive phenotype

**Figure 4:**
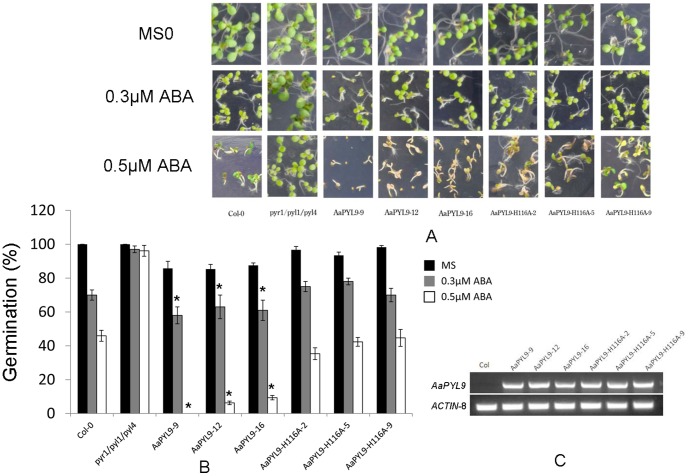
Seed-germination phenotypes of 35S::AaPYL9 and 35S::AaPYL9-H116A transgenic *Arabidopsis* plants. A: ABA-hypersensitive inhibition of germination in 35S::AaPYL9 and 35S::AaPYL9-H116A transgenic *Arabidopsis* lines compared with wild-type plants and ABA receptor triple mutant. Seeds were grown on MS medium with or without 0.3 µM ABA or 0.5 µM ABA. Photographs were taken 8 days after sowing. B: Percentage of seed germination and early seedling growth in the presence of the indicated ABA concentrations are shown. Germination percentage is determined at 8 days after seeds scored on MS (black bars), 0.3 µM ABA (dark gray bars) or 0.5 µM ABA (white bars). Data are averages ±SE from three independent experiments (n≈200 seeds per experiment). *P<0.01 (Student's t test) when comparing data for each genotype versus the wild-type under the same conditions. C: RT-PCR analysis of *AaPYL9* expression in 35S::AaPYL9 and 35S::AaPYL9-H116A transgenic *Arabidopsis* lines. RT-PCR was carried out for 30 cycles, β*-ACTIN8* transcripts were amplified as a control (lower gel).

The specific interaction between AaPYL9 and *Arabidopsis* ABI1 prompted us to test whether overexpression of AaPYL9 in *Arabidopsis* would affect ABA sensitivity in seeds and early seedling growth. The intact *AaPYL9* gene or with a point mutation *(AaPYL9-H116A)* were constructed under the control of 35S promoter and then introduced into *Arabidopsis* plants. Twenty transgenic lines of each construct were obtained and three T3 homozygous lines were chosen to be analyzed. Expression analysis showed that *AaPYL9* gene and *AaPYL9-H116A* were strongly expressed in 35S::AaPYL9 and 35S::AaPYL9-H116A transgenic *Arabidopsis* plants (Fig. 4C). *Arabidopsis pyr1/pyl1/pyl4* triple mutant lost three ABA receptors and thus was hyposensitive to ABA treatment. We investigated the germination rate of the seeds of wild type, *pyr1/pyl1/pyl4* triple mutant, three 35S::AaPYL9 transgenic *Arabidopsis* plants and three 35S::AaPYL9-H116A transgenic *Arabidopsis* plants on MS plates or MS plates supplemented with 0.3 µM or 0.5 µM ABA (Fig. 4A, 4B). All seeds germinated well on MS plates without ABA. The increase of ABA content in the plates decreased the germination rate of seeds in wild type and transgenic plants but not in the *pyr1/pyl1/pyl4* mutants, which is resistant to ABA treatment (Fig. 4B). However, the seed germination rate of the 35S::AaPYL9 transgenic *Arabidopsis* plants are below 10% while that of wild type is around 45%, showing a significant enhancement in ABA response. Because the H116A mutation abolished the interaction between AaPYL9 and AtABI1, the seed germination rate 35S::AaPYL9-H116A transgenic *Arabidopsis* plant only showed similar germination rate to wild type plants under ABA treatment, suggesting the interaction of AaPYL9 and AtABI1 is important to confer the ABA hypersensitive phenotype of the 35S::AaPYL9 plant seeds.

**Figure 5:**
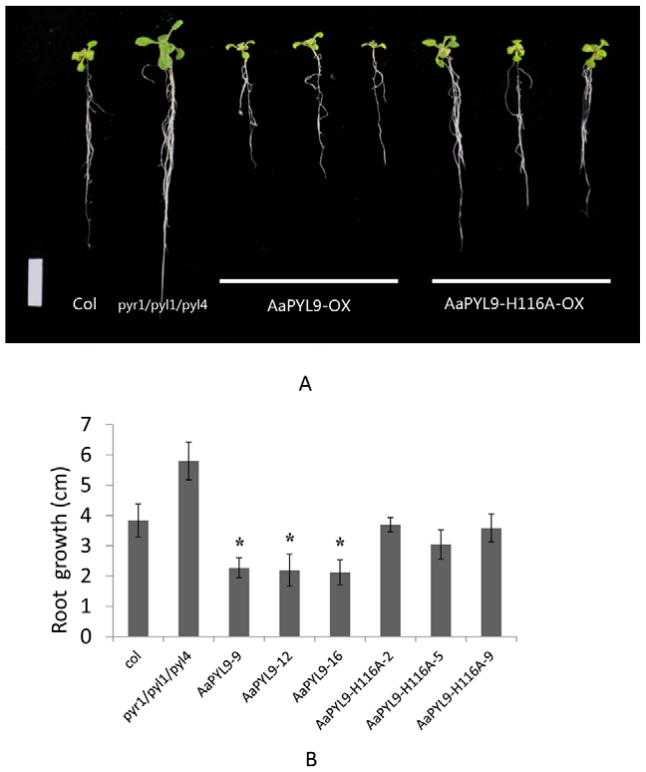
ABA hypersensitive inhibition of root growth in 35S::AaPYL9 transgenic *Arabidopsis*. A: Growth of Col-0, *pyr1/pyl1/pyl4*, three independent 35S::AaPYL9 lines (9, 12, 16) and three independent 35S::AaPYL9-H116A lines (2, 5, 9) in medium (MS) supplemented with 5 µM ABA. Photographs of representative seedlings 7 d after the transfer of 5-d-old seedlings from MS to plates supplemented with 5 µM ABA. Bar = 1 cm. B: Statistic analysis of root length in medium (MS) supplemented with 5 µM ABA. Data are averages ± SE from three independent experiments (n = 30 seeds per experiment). *P<0.01 (Student's t test) when comparing data for each genotype versus the wild-type under the same conditions.

ABA has an inhibitory role in root growth and the root growth under ABA treatment is usually adapted as a standard to evaluate plant ABA sensitivity. To fully examine the plant phenotype under ABA treatment, we plated wild type, *pyr1/pyl1/pyl4* triple mutant, three 35S::AaPYL9 transgenic *Arabidopsis* lines and three 35S::AaPYL9-H116A transgenic *Arabidopsis* lines on MS medium. After 5 days, transferred them to vertical plates supplemented with or without 5 µM ABA for 7 days. The plants grown on plates without ABA display no visible root development phenotype. However, when grown on plates supplemented with 5 µM ABA, 35S::AaPYL9 transgenic *Arabidopsis* plants showed significant reduced root length while the 35S::AaPYL9-H116A transgenic *Arabidopsis* plants did not show any statistically difference, compared with wild-type (Fig. 5A, 5B), indicating the *AaPYL9* overexpression lines were hypersensitive to ABA-mediated inhibition of root growth in *Arabidopsis*. The interaction between AaPYL9 and AtABI1 is important for its function. In addition, 35S::AaPYL9 transgenic *Arabidopsis* plants showed slight retarded growth in aerial parts of plants.

### The seeds of *AaPYL9* overexpression *Arabidopsis* are hypersensitive to salt and osmotic stress

**Figure 6:**
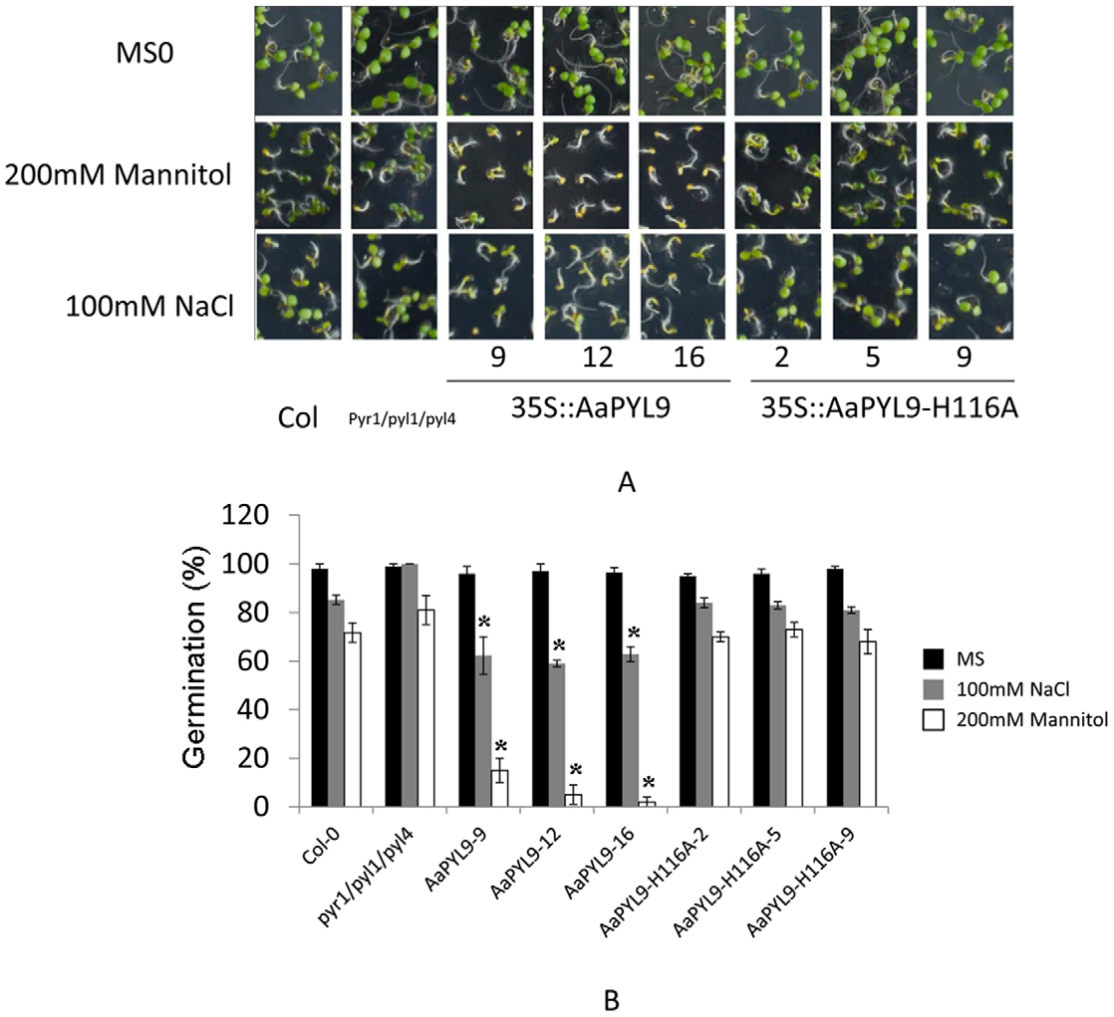
Salt stress and osmotic stress germination assays. A: Seed germination and early seedling growth in MS medium or medium supplemented with either 200 mM mannitol or 100 mM NaCl. Photographs of wild-type (Col-0), *pyr1/pyl1/pyl4* mutant, three independent 35S::AaPYL9 transgenic *Arabidopsis* lines (9, 12, 16) and three independent transgenic *Arabidopsis* 35S::AaPYL9-H116A lines (2, 5, 9) in medium supplemented with 100 mM NaCl or 200 mM mannitol. Seeds were scored 8 days after sowing. B: Percentage of 35S::AaPYL9 and 35S::AaPYL9-H116 seed germination in MS medium or medium supplemented with either 200 mM mannitol or 100 mM NaCl. Photographs were taken 8 days after sowing of seeds. Data are averages ±SE from three independent experiments (n≈200 seeds per experiment). *P<0.01 (Student's t test) when comparing data for each genotype versus the wild-type under the same conditions.

ABA perception and signaling regulates many abiotic stress responses including salt and osmotic stresses. Since 35S::AaPYL9 transgenic *Arabidopsis* plants have enhanced ABA sensitivity in germination, it is speculated that the overexpression of *AaPYL9* may also affect plants' salt and osmatic stresses at the seed germination stage. To test this, we analyzed the seed germination of 35S::AaPYL9 and 35S::AaPYL9-H116A transgenic *Arabidopsis* lines under 100 mM NaCl or 200 mM mannitol treatment (Fig. 6A, 6B). The result demonstrates that 35S::AaPYL9 seeds exhibit an enhanced degree of seed dormancy and sensitivity to inhibition of germination by exogenous ABA, and osmotic stress such as NaCl and Mannitol, suggesting that AaPYL9 is a positive regulator in ABA responsiveness in seeds.

### Analysis of ABA-responsive gene expression in transgenic *Arabidopsis* plants

**Figure 7:**
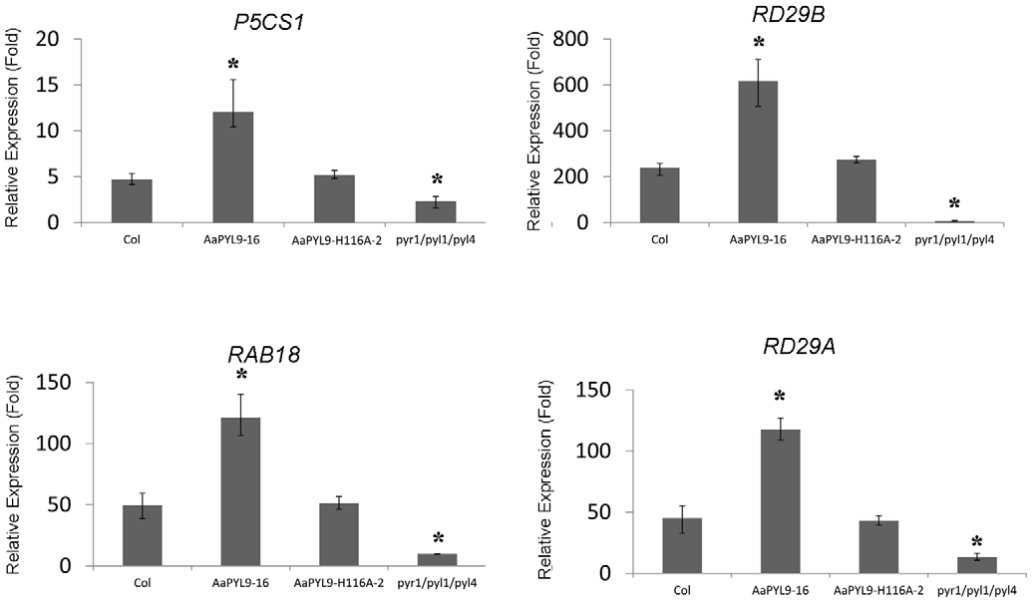
Expression analysis of ABA-regulated genes in 35S::AaPYL9 transgenic *Arabidopsis* compared to wild-type. The mRNA levels of the indicated genes are determined by Q-PCR analysis using total RNAs isolated from mock-treated *Arabidopsis* or 10 µM ABA-treated plants for 3 h. Data are averages ± SE from three independent experiments. Expression of β-*ACTIN8* was used to normalize data. *P<0.01 (Student's t test), Data are averages ± SE from three independent experiments.

To examine whether the enhancement in ABA sensitivity in transgenic plants was accompanied by altered expression of ABA-responsive genes, quantitative real-time PCR was used to monitor the expression of the ABA-inducible *RD29b* [45] and *RAB18* [46] genes. 35S::AaPYL9 transgenic *Arabidopsis* plants behave opposite to the *abi1-1* mutant, as they showed a high induction (2 to 3 fold) in the expression of *RAB18*, *P5CS1*, *RD29A*, and *RD29B* upon ABA treatment from that detected in wild-type plants (Fig. 7).

### Constitutive overexpression of *AaPYL9* in *A. annua* improves drought tolerance of *A. annua*

**Figure 8:**
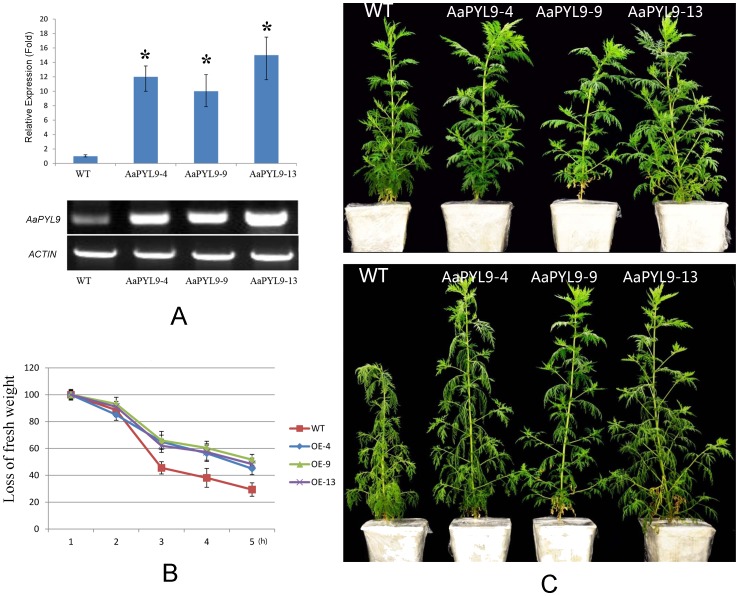
Transgenic *A. annua* plants have improved drought tolerance, reduced water loss rate under drought conditions. A: Quantitative real-time PCR and RT-PCR analyses of *AaPYL9* expression in three independent T0 35S::AaPYL9 transgenic *Artemisia* lines (4, 9, and 13) compared to wild-type. Quantitative real-time PCR was carried out via the 2-^ΔΔ^CT method using the *A. annua ACTIN* gene as an internal control, *P<0.01 (Student's t test). The values are presented as the average and ±SE from three independent experiments. RT-PCR is carried out for 25 cycles. *A. annua ACTIN* gene transcripts were amplified as a control (lower gel). B: Loss of fresh weight measured in approximately 1 g fresh leaves of either wild-type plant or three independent T0 35S::AaPYL9 transgenic *Artemisia* lines (4, 9, and 13). Data values represent one of three independent experiments with similar results. C: Whole plant transpiration assay. Photograph shows the drought phenotype of three independent 35S::AaPYL9 T0 transgenic *Artemisia* lines (4, 9, and 13) compared with wild-type.

To determine the AaPYL9 is a real functional ABA receptor in *A. annua*, a gain-of-function *A. annua* was generated. Twelve independent transformants were selected in hygromycin-containing medium and further confirmed for the transgenic status by PCR (Fig. S3). Three T0 lines were chosen to be further analyzed. The expression level of *AaPYL9* in transgenic *A. annua* plants was >10–20 times higher than that in wild type, showing that the transgenic plants highly express the *AaPYL9* constitutively (Fig. 8A). Under normal growth conditions, the transgenic plants did not show obvious morphological or developmental abnormalities (Fig. 8C, upper). However, when subjected to drought stress for 2 weeks, the wild type plants showed more wilt than transgenic ones (Fig. 8C lower). ABA triggers stomatal closure and reduces water loss under drought conditions. *AaPYL9* overexpression lines showed lower rates of transpiration than wild-type plants under drought conditions (Fig. 8B). Recently, the *Arabidopsis* PYR/PYL/RCAR receptors were reported to be involved in regulation of stomatal closure [47], [48]. Therefore, the stomatal response analyses were performed with *AaPYL9* overexpression line, AaPYL9-9, compared to wild type *Artemisia*. The AaPYL9-9 line showed an ABA hypersensitive phenotype in ABA -induced stomatal closing (Fig. S4). These results suggest that overexpression *AaPYL9* in *Artemisia* improves dought tolerance, induces stomatal closure, and reduces rates of transpirtion.

### Constitutive *AaPYL9* overexpression in *Artemisia* up-regulates the expression of Artemisinin biosynthetic genes and enhances Artemisinin production when sprayed with exogenous ABA

**Figure 9:**
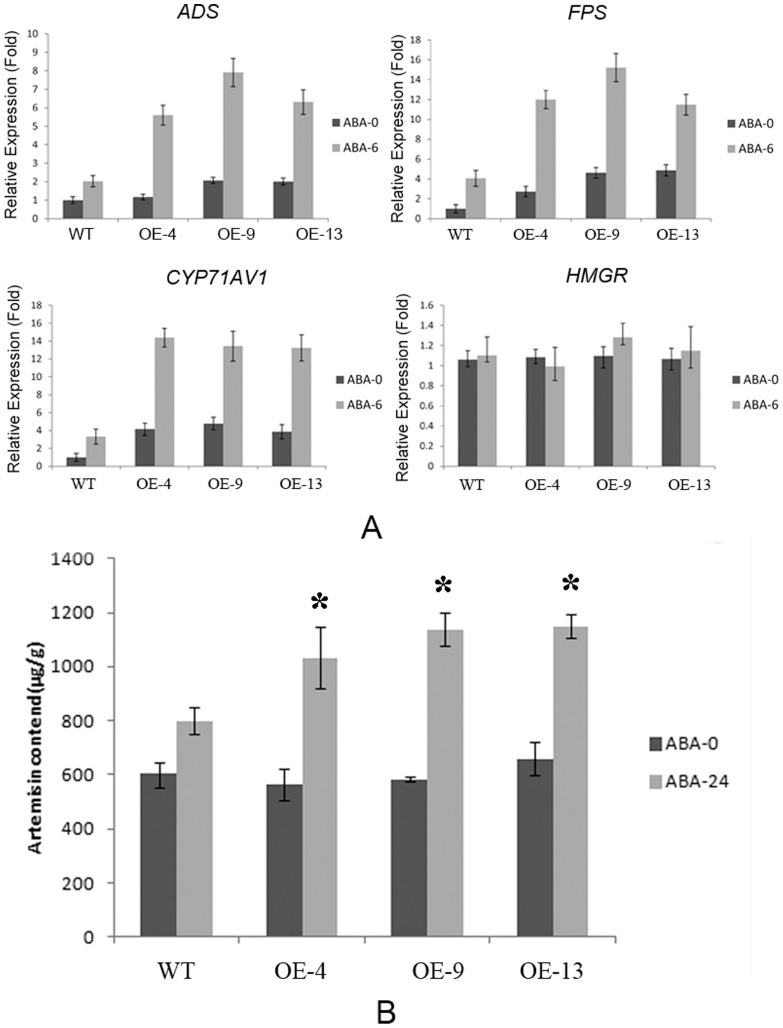
Overexpression *AaPYL9* in *Artemisia* increased the expression of artemisinin biosynthesis genes and artemisinin contents. A: One-month-old wild-type and three independent T0 35S::AaPYL9 transgenic *Artemisia* plants are treated with 10 µM ABA solution for 6 h, and the young leaves were used in quantitative RT-PCR analysis. Data represent means ±SE from three replicates. *P<0.01 (Student's t test). B: Artemisinin content of three independent T0 35S::AaPYL9 transgenic *Artemisia* lines and wild-type treated by 10 µM ABA. The wild-type showed an increase of 33% in artemisinin content, while 35S::AaPYL9 line was increase of 74%–95%. The Artemisinin content was measured three times by HPLC-ELCD. *P<0.01 (Student's t test).

Exogenous ABA spray can increase the production of artemisinin by inducing the expression of key genes such as *ADS*, *FPS* and *CYP71AV1* in artemisinin biosynthesis pathway [40]. To examine whether constitutive overexpression of *AaPYL9* could enhance artemisinin production when sprayed with exogenous ABA, we compared the expression of several artemisinin biosynthetic key genes, *HMGR*, *ADS*, *FPS*, and *CYP71AV1*, in *AaPYL9* overexpression lines with that of wild-type, before ABA-treatment and after ABA-treatment for 6 h. In both wild-type and transgenic lines, the expression of *ADS*, *FPS* and *CYP71AV1* but not *HMGR* was enhanced after ABA-treatment (Fig. 9A). However, transgenic lines showed a significant higher induction in the expression of *ADS*, *FPS* and *CYP71AV1* upon ABA treatment than wild-type plants (Fig. 9A). At the same time, we detected the content of artemisinin. Before ABA treatment, the artemisinin content of wild-type *Artemisia* as well as 35S::AaPYL9 transgenic lines was approximately 600 (µg/g). However, when we analyzed ABA treated leaves, the artemisinin content of wild-type *Artemisia* was increased by 33%, while the artemisinin content of 35S::AaPYL9 transgenic lines was increased by 74%∼95% (Fig. 9B). Therefore, overexpression *AaPYL9* in *Artemisia* will enhance the ABA sensitivity upon ABA treatment, and improved the artemisinin content.

## Discussion

After the discovery of ABA receptors, PYR/PYL/RCAR gene family, in *Arabidopsis* [21]–[23], orthologues of these genes were identified in genomes ranging from mosses to crops, suggesting that ABA signaling components may have arisen as plants evolved from aquatic organisms to terrestrial organisms [19], [32], [49]–[51]. Some of them have been identified in *Oryza sativa* [52], *Nicotiana tabacum* [50], *Vitis vinifera* [51], and *Fragaria ananassa* [49], [53] by experimental analysis. And Saavedra et al. demonstrated a PP2C from *Fagus sylvatica* which can interact with AtPYL8, and function in *Arabidopsis* [54]. In this study, we identified an ABA receptor in medicinal plant *Artemidisa annua* L., AaPYL9, which was highly conserved in amino acid sequence with *Arabidopsis* AtPYL9. AaPYL9 can interact with AtABI1 in yeast two-hybrid system and *in planta*, and ectopic overexpression of *AaPYL9* in *Arabidopsis* causes ABA-hypersensitive phenotype. These results demonstrate that PYR/PYL/RCAR and PP2C are highly conserved in different species in terms of biological function, sequence, and functional mechanisms. Kim et al reconstitutes rice OsPYL/RCAR5, OsPP2C30, SAPK2, and OREB1 in *Arabidopsis* protoplasts and demonstrates those components are a minimal functional unit for ABA sensing, ABA signal transduction, and the activation of ABRE-dependent transcription in rice [52]. Thus, the ABA signal transduction pathway mediated by PYR/PYL/RCAR and PP2C is highly conserved among evolutionarily distant plant species. It would be of interest to clone other key components in ABA signaling from *Artemisia*, such as PP2Cs, SnRKs, to constitute a complete ABA signaling pathway in this medicinal plant.

The discovery of PYR/PYL/RCAR proteins has led to a series of crystallographic studies [23], [25]–[28], [55]. All reports confirm the importance of the helix-grip fold in ABA binding. The PYR/PYL/RCAR proteins have two loops and two pairs of β-strands, both are important for ABA perception. The β3–β4 loop is called gate while the β5–β6 loop is called latch [25]. It was found that PYR1R157H, PYR1P88S and PYR1S152L were three pyrabactin-insensitive mutants, and yeast two-hybrid result showed PYR1R157H can interact with HAB1 in the existence of exogenous ABA, but PYR1P88S and PYR1S152L cannot [22]. Melcher et al. demonstrates that the latch residue H115 is not important for ligand binding in the context of PYR1 itself, but is critical for the receptor to relay the ABA binding signal to PP2Cs and downstream signaling effectors [25]. The deduced AaPYL9 protein three-dimensional structure contained two loops and two pairs of β-strands, which was similar with PYR/PYL/RCAR in *Arabidopsis*. Therefore, we present two point-mutations in the AaPYL9 SGLPA gate (P89S), and the latch (H116A), both of them weakened the interaction with AtABI1 in yeast two-hybrid assays. Furthermore, we test the function of AaPYL9H116A mutant *in planta*, the seed germination and root elongation of 35S::*AaPYL9*-H116A were similar with wild-type. These results demonstrate that the AaPYL9 gate residue P89 and latch residue H115 are critical for interaction with AtABI1 and downstream signaling. Together, the ABA receptor of *Artemisia* has similar structural basis for ABA signaling with *Arabidopsis*, providing useful information for the evolution of ABA signaling perception and transduction.

In addition, ABA signaling also regulates plant abiotic stress adaptation, development and secondary metabolites production. Previous studies show that plant abiotic stress tolerance has been improved in the transgenic plants overexpressing ABA receptors [54], [56]. In this study, overexpression of *AaPYL9* in *Arabidopsis* increased the plants' sensitivity to ABA and thus reduces the seedling growth under ABA, osmotic and salt stress treatments. In agreement well with this, transgenic *Artemisia* plants overexpressing *AaPYL9* also shows drought tolerant phenotype, which may be of great value to the growth of this important medicinal plant. ABA affects the biosynthesis of several secondary metabolites, such as that of terpenoid indole alkaloids in *Catharanthus roseus* [57] and anthocyanins in *Arabidopsis* [58], Maize [59], and Grape berry [60], [61]. Recently, Lackman et al. reported ABA receptor NtPYL4 regulated metabolic reprogramming in tobacco [50]. Furthermore, our previous results showed artemisinin content could be increased significantly when sprayed with exogenous ABA to the *Artemisia* plants before harvesting [40]. Accordingly, we tested the artemisinin content in *AaPYL9*-OX *Artemisia* compared to wild type. The results demonstrated that overexpression of *AaPYL9* caused increased artemisinin accumulation when treated with ABA. Although showing similar artemisinin content under normal growth condition, AaPYL9-OX plants had significantly more increase (∼1.7 fold) of artemisinin content after ABA treatment than wild-type (∼1.3 fold). The more increase of artemisinin production may be due to the more receptors in the transgenic plants which sense the exogenous ABA. Under normal growth condition, the ABA receptors in wide type may be excess and in the rest form, and the ABA concentration was quite low, thus overexpression of ABA receptor will not increase the sensitivity to the endogenous ABA. But upon ABA treatment, ABA levels increased to achieve marked phenotype. Overexpression of *AaPYL9* in *Arabidopsis*, improved ABA signaling in the plant under ABA treatment, which can be revealed by expression of ABA responsive genes, such as *P5CS1*, *RD29A*, *RD29B*, *RAB18* (Fig. 7). In *Artemisia*, the increased ABA signaling finally induces the expression of the key genes in artemisinin production. Previous results also showed that salt and osmotic treatment increased artemisinin production. It would be interesting to see if those abiotic stresses can increase artemisinin more in the *AaPYL9*-OX plants.

In this study, we cloned and analyzed AaPYL9, an ABA receptor orthologue from medicinal plant, *A. annua*. Sequence analysis, structure prediction and ABA binding in yeast assay support that AaPYL9 is an ABA receptor. Protein-protein interaction assay shows that AaPYP9 can interact specifically with AtABI1. PYR/PYL/RCAR proteins act redundantly in several plant species, such as *Oryza sativa* [52], *Vitis vinifera* [51], *Fragaria ananassa* [49]. In *Arabidopsis*, until now, no single PYR/PYL/RCAR mutant shows an ABA hyposensitive phenotype but rather at least triple mutant is needed to give a reasonable ABA hyposensitive phenotype. A systematic analysis in bryophyte, lycophyte and angiospem revealed that vascular plants had more than 10 PYR/PYL/RCAR homologues [32]. In *Artemisia*, based on the current database, there are at least 8 PYR/PYL/RCAR homologues, and no mutants are available. Besides, considering the redundancy of PYL genes in vascular plants, it is difficult to get knock-down lines with observed ABA hyposensitive phenotype. Therefore, we just overexpressed *AaPYL9* in *Artemisia* instead of constructing *AaPYL9* knock-out or knock-down plants. *AaPYL9* overexpressing plants increased ABA sensitivity, resulting in more tolerance to abiotic stress and more artemisinin production. This is very important for *Artemisia* cultivate, because most of *Artemisia*, which can be used for extract artemisinin, is grown on mountain area of southern China, where drought is the major threat for this medicinal plants. Thus, *AaPYL9* would be of great value in both ABA signaling study and genetic engineering in the important medicinal plant, *Artemisia*.

## Materials and Methods

### Chemicals

(+-)Abscisic acid was obtained from Sigma-Aldrich (http://www.sigmaaldrich.com), the other chemicals were purchased from China National Medicines Corporation Ltd (http://www.sinopharm.com/).

### Plant material and ABA treatments

*A. annua* used in this study was obtained from Chongqing, China. Plants of *A. annua* were grown in the greenhouse with 16/8-h light/dark photoperiod at 26°C. For ABA, NaCl treatment, plants of 15-day-old *A. annua* were sprayed with ABA solution (10 µM) or NaCl solution (200 µM) for 1, 3 and 6 h. For drought stress, 15-day-old *A. annua* were transferred from water-saturated soil to filter paper for 1, 3 and 6 hours. The *Arabidopsis* (*Arabidopsis thaliana*) wild-type and transgenic plants used in this work were Col-0 ecotype, and they were grown in pots in a growth chamber under 24°C and 16-h-light/8-h-dark photoperiod at 80 to 100 mE m^−2^ s^−2^. For *in vitro* culture, seeds were surface sterilized in 10% sodium hypochlorite solution and 0.01% Triton X-100 for 5 min, and washed three times in sterile distilled water. After conducted during 3 d at 4°C, the seeds were sown on plates containing MS solid medium composed of MS basal salts and 20% sucrose solidified with 0.6% agar with pH 5.7. Plates were sealed and incubated in a controlled-environment growth chamber. Seedlings of the *A. annua* were obtained from 4 weeks-stratified seeds sown in a controlled environment chamber under the same condition of *Arabidopsis*.

### Isolation of the full-length cDNA clone

A cDNA library constructed in the vector using poly (A+) RNA from leaves of 4-month-old *Artemisia* grown in greenhouse. Randomly picked clones were sequenced with an ABI 3700 DNA Sequencer, and sequences were assembled and edited using PHRED/PHRAP. The non-redundant unigenes (contigs and singlets) were subjected to similarity searches against the GenBank non-redundant (nr) protein databases using the BLASTX algorithm with default parameters, and then the AaPYL9/RACR1 sequence was identified.

### Point mutagenesis of AaPYL9

Point mutation of AaPYL9 was performed using overlapping PCR strategy. To make P89S mutation, overlapped fragments of AaPYL9 were amplified using KOD-plus DNA polymerase (TOYOBO) using two pairs of primers: AaPYL9-F and AaPYL9-P89S-R, AaPYL9-P89S-R and AaPYL9-R. The PCR products were separated by 1.2% agarose gel and the DNA was extracted using a Gel extraction kit (Invitrogen). Purified PCR products were mixed, and 1 µl of the mixed DNA was used as the template in PCR amplification by KOD-plus DNA polymerase using AaPYL9-F and AaPYL9-R primers for Y2H and overexpression vector construction. The same method was used to make H116A mutation. All the mutations were checked before use.

### Vector Construction and Plant Transformation

To generate the constructs, the open reading fragment of the AaPYL9 cDNA was amplified by PCR and cloned into pENTR vector (Invitrogen) and sequenced. For yeast two-hybrid screening, pENTR*-AaPYL9* was transferred to the bait vector pDEST32 by Gateway LR recombination reaction (Invitrogen). ABI1, ABI2, HAB1 and HAB2 from *Arabidopsis* were cloned into pENTR vector and then transferred to the split vector pDEST22. To prepare vectors for AaPYL9 localization, the plasmid pEarleyGate104-35S::YFP::*AaPYL9* was generated by LR recombination reaction (Invitrogen). To overexpressing *AaPYL9* in *Arabidopsis* we transferred the pENTR-*AaPYL9* to the pEarleyGate101-35S::*AaPYL9*. The similar method was used to make AaPYL9-P89S and AaPYL9-H116A overexpression and Y2H vectors. BiFC vectors pEarleyagte201-YN and pEarleygate202-YC were kindly provided by Professor Steven J. Rothstein. pENTR-*AaPYL9* was transferred into pEarleyagte202-YN and pENTR-AtABI1 was transferred into pEarleyagte201-YC by Gateway LR recombination reaction (Invitrogen), which were named pEG202yc-*AaPYL9* and pEG201yn-*AtABI1*. pEG101-*AaPYL9*, pEG101-AaPYL9-P89S and pEG101-AaPYL9-H116A constructs were introduced into *Arabidopsis* using a flower dipping method [62]. The full-length coding region of AaPYL9 was amplified by PCR with sticky *EcoR*I and *Bam*HI ends, and inserted into the binary vector PHB under the control of the double CaMV 35S promoter. The resulting construct (PHB-*AaPYL9*) was introduced into *Agrobacterium tumefaciens* strain EHA105. *A. annua* transgenic plants were generated as previously described [63]. Generally, seeds of *A. annua* were surface-sterilized by treatment with 75% (v/v) ethanol for 1 min, followed by treatment with 20% (v/v) NaOCl (sodium hypochlorite) containing Tween-20 for 20 min, and washed three to four times with distilled water and finally sown in germination medium MS_0_ basal medium (supplemented with 3% sucrose, 0.4% phytagel and adjusted to pH 5.8) with a photoperiod of 16 h light/8 h dark and light at 8000 lux (metal halide source) at 25°C. After two weeks, the germinated seedlings were collected and the leaves were cut into 0.5-cm-diameter discs and used as the explants in *Agrobacterium*-mediated co-cultivation. After hygromycin selection in the selective medium MS_1_ (MS_0_+2.5 mg/l *N* ^6^-benzoyl adenine+0.25 mg/l naphthalene-1-acetic acid+50 mg/l hygromycin+250 mg/l carbenicillin), the hygromycin-resistant plantlets were regenerated and then transferred into rooting medium MS_2_ (half-strength MS_0_+250 mg/l carbenicillin). After one month, the rooted plantlets were transferred into soil into pots in the growth chamber.

### Protoplast Analysis

The expression vector pEarleyGate 104-35S::*YFP*::*AaPYL9* was transferred into *Arabidopsis* protoplasts. Preparation and analysis of *Arabidopsis* protoplasts was performed as described by Moes et al. [64].

### Yeast Two-Hybrid assay

*AaPYL9* was cloned in a bait vector pDEST32, and at the same time *ABI1*, *ABI2*, *HAB1* and *HAB2* from *Arabidopsis* were cloned in a split vector pEDST22. We co-transformed the specific pairs of BD and AD constructs into the yeast strain AH109 by the lithium acetate method, and the transformants were spread on media plates that lacked leu, trp (SD/-T-L). Five independent 3-d-old colonies with the same size were picked up and diluted into 100 µl 1×TE, and aliquots of 10 µl yeast cells were dropped on selective medium that lacked trp, leu, his(SD/-T-L-H) and lacked trp leu, his, ade(SD/-T-L-H-A) supplemented with or without 10 µM ABA.

### BiFC Assay

For BiFC assay, *Arabidopsis* protoplasts were transfected with plasmid pEG202yc-AaPYL9 and pEG201yn-AtABI1 DNAs (10–20 µg). YFP fluorescence was evaluated using a Fluoview FV300 CLSM system (Olympus Ltd) 3 h after transfection.

### Germination Assay

To measure ABA sensitivity, approximately 200 seeds each from wild-type (Col-0), three independent 35S::AaPYL9 transgenic *Arabidopsis* lines (9, 12, 16) and 35S::AaPYL9-H116A transgenic *Arabidopsis* lines (2, 5, 9) as well as ABA receptor triple mutant *pyr1/pyl1/pyl4 Arabidopsis* were sterilized and plated on solid medium composed of MS basal salts, 2% Glc, and different concentrations of ABA (0.3 and 0.5 µM). After 4 days of stratification at 4°C in the dark, plates were incubated at 22°C under 16-h light/8-h dark cycle. All seeds were harvested on the same day in identical environmental conditions. In order to score seeds germination, the seeds that formed green shoots were considered to be germination. The percentage of seeds was determined in all assays after 8 days of sown. Photographs were taken 8 days after sown. Each value represents the average germination percentage of approximately 200 seeds with the ±SE value of three replicates.

To determine sensitivity to inhibition of germination by low osmoticum and salt, Approximately 200 seeds each from wild-type (Col-0), three independent 35S::AaPYL9 transgenic *Arabidopsis* lines (9, 12, 16) and 35S::AaPYL9-H116A transgenic *Arabidopsis* lines (2, 5, 9) as well as ABA receptor triple mutant *pyr1/pyl1/pyl4 Arabidopsis* were sterilized and plated on the MS medium and MS medium supplemented with 200 mM mannitol or 100 mM NaCl, respectively. Seedling establishment was scored as the percentage of seeds that developed green expanded cotyledons after 7 days of sowing. Data are means ±SE from three independent experiments (n≈200 seeds per experiment).

### Root Growth Assay

To measure ABA hypersensitive inhibition of root growth, approximately 20 seedlings per experiment were sterilized and plated on MS medium for 4 days. Then, plants were transferred a new plate containing MS medium supplemented with or without 5 µM ABA. Photographs were taken after 7 d of transferring, and quantitatively analyzed using NIH IMAGE J software (http://rsb.info.nih.gov/nih-image/). Data are averages ±SE from three independent experiments.

### Real-Time PCR Assay

For analysis of ABA-responsive gene expression in transgenic *Arabidopsis*, *Arabidopsis* leaves from 10-day-old seedlings grown on MS agar plates were used for mock treated or treated with 10 µM ABA during 3 h. For analysis of artemisinin biosynthetic genes induced by exogenous ABA, the whole plant of *Artemisia* was sprayed with 10 µM ABA solution during 6 h. Plant material was collected and frozen in liquid nitrogen. Total RNA for quantitative reverse transcription (RT)-PCR was extracted using RNA Extract Kit as described by the manufacturer (Tiangen). Real-time PCR was performed in a Bio Rad MJ (Bio Rad). Amplification was carried out with Brilliant SYBR Green QPCR MasterMix (Takara) according to the manufacturer's instructions. The thermal profile for SYBR Green real-time PCR was 95°C for 2 min, followed by 40 cycles of 95°C for 15 s and 60°C for 1 min. The expression of all genes was normalized against the expression of the endogenous control gene (β-*ACTIN8*). All experiments were repeated at least three times.

### Transpiration and stomatal aperture Assay

For the transpiration assay, the loss of fresh weight of approximate 1 g leaves at the same developmental stage was measured at room temperature after the indicated periods of time. To score ABA-induced stomatal closing, *Artemisia* leaves were incubated for 2 h in a solution containing 10 mM KCl, 0.2 mM CaCl2, and 10 mM MES-KOH (pH 6.15) under white light for stomata fully open. Then, they were incubated for 2 h in the same buffer supplement or not with 10 µM ABA. Stomatal apertures were measured from images obtained by using Nikon ECLIPSE TS100 with IMAGEJ software. Each value represents the average aperture approximately 40 stomata with the ±SE value of three replicates.

### Quantification of Artemisinin Using HPLC-ELSD (HPLC–evaporative light-scattering detection)

Leaves of *A. annua* were sprayed by 10 µM ABA, and collected immediately which were defined as time 0, and leaves collected after 24 h were defined as time 24. The method of quantification of artemisinin was performed as previously described [63].

## Supporting Information

Figure S1 **Expression analysis of** ***AaPYL9*** **in different tissues and under ABA, NaCl and drought treatment.** A, Expression analyses of *AaPYL9* in different *Artemisia annua* L. tissues. Real-time PCR analysis of total RNA isolated from *Artemisa annual* L. untreated tissues. Quantitative RT-PCR was performed for the organ specific expression analysis and the internal control gene *ACTIN* was used to show the normalization of the amount of templates in PCR. B, Real-time PCR analysis of *AaPYL9* expression in *Artemisia* treated with 10 µM ABA (gray bar) or treated with mock (black bar). C, Real-time PCR analysis of *AaPYL9* expression in *Artemisia* treated with 200 µM NaCl (gray bar) or treated with mock (black bar). D, Real-time PCR analysis of *AaPYL9* expression in *Artemisia* under drought condition. Students' t test showed that the expression of *AaPYL9* have no significant change when treated with 10 µM ABA, 200 µM NaCl, as well as under drought condition. Data represent means ±SE from three replicates.
(TIF)

Figure S2 **Yeast two-hybrid assay for AaPYL9- interacting proteins.** Yeast two-hybrid interaction test of AaPYL9 with AtABI2, AtHAB1 and AtHAB2. AaPYL9 could not interact with AtABI2, AtHAB1, AtHAB2 in selective medium (SD/-T-L-H;SD/-T-L-H-A; SD/-T-L-H-A+10 µM ABA).
(TIF)

Figure S3 **PCR analyses of independent transgenic** ***A. annua*** **plants.** PCR analysis for the presence of *AaPYL9* overexpression constructs in *A. annua* plants. WT: wild-type *A. annua* plant (negative control); P: Positive control (PHB-AaPYL9 plasmid). The forward primer used in this PCR analysis was located in 35S promoter, while reverse primer was located in *AaPYL9* open reading fragment.
(TIF)

Figure S4 **Stomata of the** ***AaPYL9*** **overexpression** ***Artemisia*** **showed increased ABA response.** A: photographs of stomatal of 35S::AaPYL9 transgenic *Artemisia* (9) and wild-type plants in the presence or absence of ABA. B: Average stomatal aperture of *AaPYL9* overexpression transgenic *Artemisia* and wild-type plants in the presence or absence of ABA. Stomata aperture of the AaPYL9-9 plants significantly decreased compared with wild type. *P≤0.01 (student's t test). Data are averages ± SE from three independent experiments (n≈40 stomata per experiment).
(TIF)
