# Supplementary figures and images for: Overexpression of the Artemisia Orthologue of ABA Receptor, AaPYL9, Enhances ABA Sensitivity and Improves Artemisinin Content in Artemisia annua L
Source: PLoS One. 2013 Feb 20;8(2):e56697. doi: 10.1371/journal.pone.0056697 (PMC3577733; doi:10.1371/journal.pone.0056697)

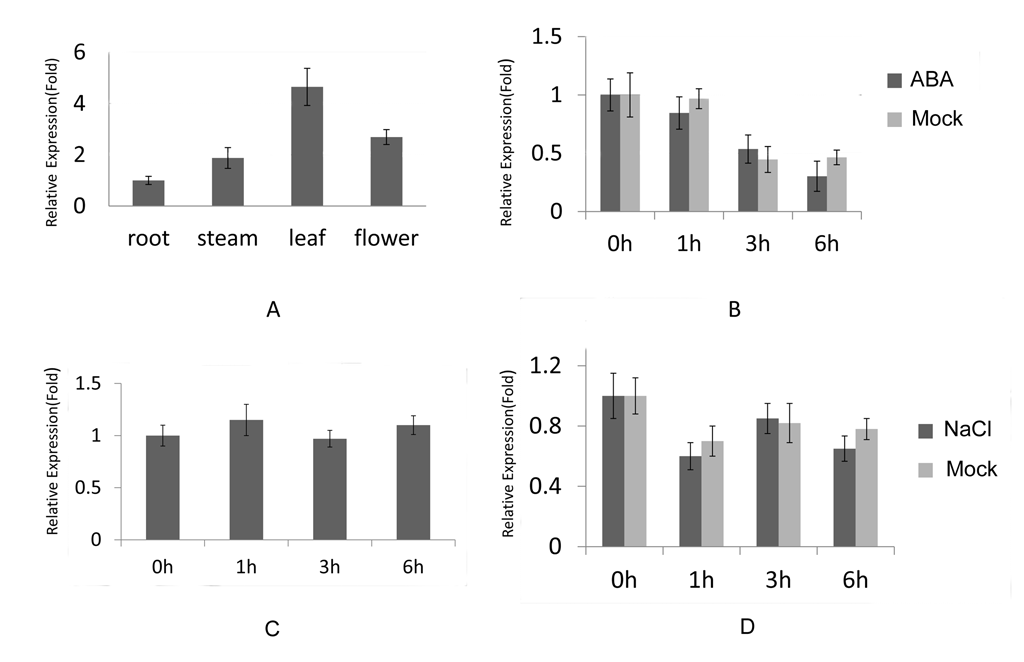

Supplement: Figure S1 — Expression analysis of AaPYL9 in different tissues and under ABA, NaCl and drought treatment. A, Expression analyses of AaPYL9 in different Artemisia annua L. tissues. Real-time PCR analysis of total RNA isolated from Artemisa annual L. untreated tissues. Quantitative RT-PCR was performed for the organ specific expression analysis and the internal control gene ACTIN was used to show the normalization of the amount of templates in PCR. B, Real-time PCR analysis of AaPYL9 expression in Artemisia treated with 10 µM ABA (gray bar) or treated with mock (black bar). C, Real-time PCR analysis of AaPYL9 expression in Artemisia treated with 200 µM NaCl (gray bar) or treated with mock (black bar). D, Real-time PCR analysis of AaPYL9 expression in Artemisia under drought condition. Students' t test showed that the expression of AaPYL9 have no significant change when treated with 10 µM ABA, 200 µM NaCl, as well as under drought condition. Data represent means ±SE from three replicates. (TIF) [file pone.0056697.s001.tif]

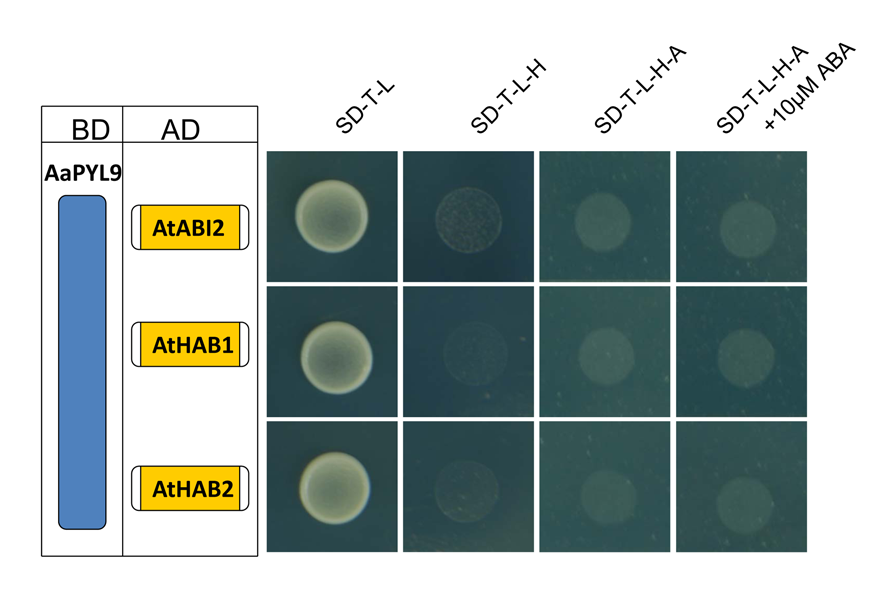

Supplement: Figure S2 — Yeast two-hybrid assay for AaPYL9- interacting proteins. Yeast two-hybrid interaction test of AaPYL9 with AtABI2, AtHAB1 and AtHAB2. AaPYL9 could not interact with AtABI2, AtHAB1, AtHAB2 in selective medium (SD/-T-L-H;SD/-T-L-H-A; SD/-T-L-H-A+10 µM ABA). (TIF) [file pone.0056697.s002.tif]

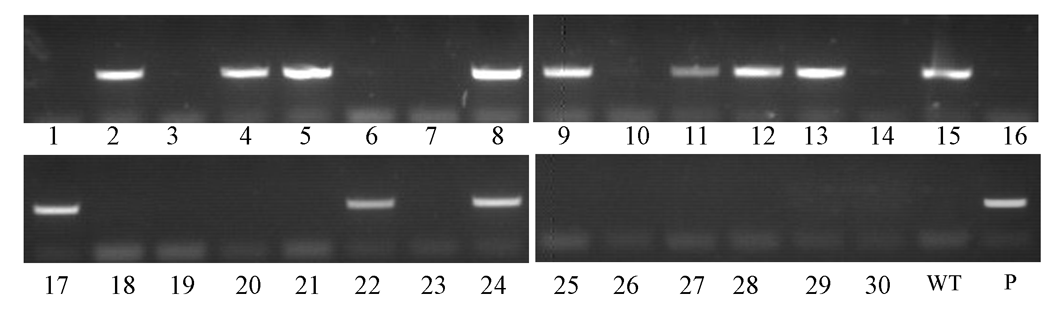

Supplement: Figure S3 — PCR analyses of independent transgenic A. annua plants. PCR analysis for the presence of AaPYL9 overexpression constructs in A. annua plants. WT: wild-type A. annua plant (negative control); P: Positive control (PHB-AaPYL9 plasmid). The forward primer used in this PCR analysis was located in 35S promoter, while reverse primer was located in AaPYL9 open reading fragment. (TIF) [file pone.0056697.s003.tif]

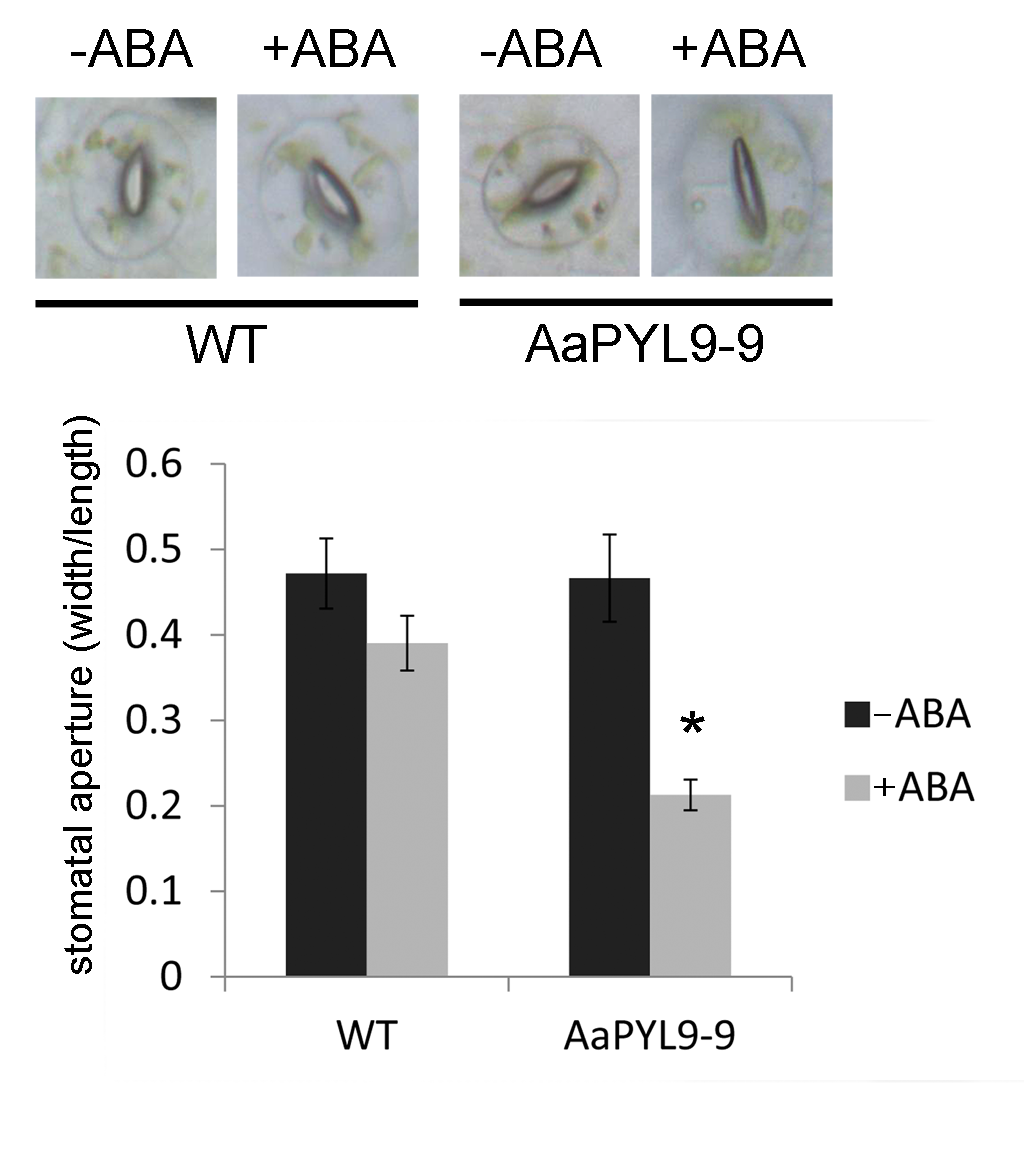

Supplement: Figure S4 — Stomata of the AaPYL9 overexpression Artemisia showed increased ABA response. A: photographs of stomatal of 35S::AaPYL9 transgenic Artemisia (9) and wild-type plants in the presence or absence of ABA. B: Average stomatal aperture of AaPYL9 overexpression transgenic Artemisia and wild-type plants in the presence or absence of ABA. Stomata aperture of the AaPYL9-9 plants significantly decreased compared with wild type. *P≤0.01 (student's t test). Data are averages ± SE from three independent experiments (n≈40 stomata per experiment). (TIF) [file pone.0056697.s004.tif]
